# RunScribe Sacral Gait Lab™ Validation for Measuring Pelvic Kinematics during Human Locomotion at Different Speeds

**DOI:** 10.3390/s23052604

**Published:** 2023-02-27

**Authors:** Emilio J. Ruiz-Malagón, Felipe García-Pinillos, Alejandro Molina-Molina, Víctor M. Soto-Hermoso, Santiago A. Ruiz-Alias

**Affiliations:** 1 Department of Physical Education and Sports, Faculty of Sport Sciences, University of Granada, 18071 Granada, Spain; 2 Sport and Health University Research Institute (iMUDS), University of Granada, 18071 Granada, Spain; 3 Department of Physical Education, Sports and Recreation, Universidad de La Frontera, Temuco 4811230, Chile; 4 Campus Universitario, Universidad San Jorge, Villanueva de Gállego, 50830 Zaragoza, Spain

**Keywords:** sacrum, vertical oscillation, tilt, obliquity, rotation

## Abstract

Optoelectronic motion capture systems are considered the gold standard for measuring walking and running kinematics parameters. However, these systems prerequisites are not feasible for practitioners as they entail a laboratory environment and time to process and calculate the data. Therefore, this study aims to evaluate the validity of the three-sensor RunScribe Sacral Gait Lab™ inertial measurement unit (IMU) in measuring pelvic kinematics in terms of vertical oscillation, tilt, obliquity, rotational range of motion, and the maximum angular rates during walking and running on a treadmill. Pelvic kinematic parameters were measured simultaneously using an eight-camera motion analysis system (Qualisys Medical AB, GÖTEBORG, Sweden) and the three-sensor RunScribe Sacral Gait Lab™ (Scribe Lab. Inc. San Francisco, CA, USA) in a sample of 16 healthy young adults. An acceptable level of agreement was considered if the following criteria were met: low bias and SEE (<0.2 times the between-subject differences SD), almost perfect (r > 0.90), and good reliability (ICC > 0.81). The results obtained reveal that the three-sensor RunScribe Sacral Gait Lab™ IMU did not reach the validity criteria established for any of the variables and velocities tested. The results obtained therefore show significant differences between the systems for the pelvic kinematic parameters measured during both walking and running.

## 1. Introduction

Human movement is a complex task that requires correct intersegmental coordination and human locomotion is concerned in particular with the forward propulsion of the body [1]. In this sense, pelvic kinematics play an important role in both maximizing athletic performance and in minimizing the risk of injury in runners during human locomotion [1,2,3]. Hence, it was suggested that their analysis in all the planes (sagittal, frontal, and transverse) could reveal important information for practitioners [4,5,6,7]. However, the accurate measurement of pelvic kinematics constitutes an essential element for clinicians or trainers working in human locomotion, in order to avoid misinterpretations [8].

Optoelectronic motion capture systems are the gold standard for measuring the kinematics parameters of walking and running. In fact, these systems have improved in recent years and the current measurement error can be less than <0.5 mm [9]. However, optoelectronic motion capture systems require laboratory settings in addition to the time and knowledge for data processing, making them unfeasible systems for some practitioners [10,11]. In response to these limitations, Inertial Measurement Units (IMUs) are a low-cost alternative that allows data to be collected in the field [12]; reports are provided immediately [13]. Nowadays, there is a wide variety of IMUs that allow kinematic monitoring of various joints in everyday tasks. Different levels of agreement have been reported according to the task performed and the joint or axis measured [14,15,16,17]. Therefore, a more detailed analysis of the new IMUs entering the market is required.

The RunScribe™ IMU system (Scribe Labs Inc., San Francisco, CA, USA) is an example of such a device widely used in the field of human locomotion analysis, consisting of two IMU devices (one on each foot). Each IMU is based on a nine-axis (three-axis gyroscope, three-axis accelerometer, three-axis magnetometer) with a sampling rate of 500 Hz. Both allow the measurement of several spatiotemporal parameters of running [18] and foot-strike patterns [19] for each foot which have previously been validated. The hardware device weighs 15 g and measures 35 × 25 × 7.5 mm. Recently, the same manufacturer launched a three-sensor system (RunScribe Sacral Gait Lab™) that includes an additional device to be placed on the runner’s sacrum that allows the measurement of the pelvic kinematics in the frontal, sagittal, and transverse planes synchronously with the two previous sessions that were placed on both feet. This would allow the already validated spatiotemporal parameters of the feet [18] to be combined with the pelvic motion, providing health and sports professionals with a complete analysis of their patients and athletes. Therefore, the aim of this study is to examine the validity of the RunScribe Sacral Gait Lab™ to measure pelvic kinematics in terms of vertical oscillation, tilt, obliquity, rotational range of motion, and maximal angular rates during walking and running on a treadmill.

## 2. Materials and Methods

### 2.1. Experimental Design

Pelvic kinematic parameters were simultaneously recorded using an eight-camera motion analysis system (Qualisys Medical AB, Sweden) and the three-sensor RunScribe Sacral Gait Lab™ IMU (Scribe Lab. Inc. San Francisco, CA, USA) during a walking and running treadmill protocol (WOODWAY Pro XL, Woodway, Inc., Waukesha, WI, USA). To acclimate to the treadmill, participants began with a 10-min warm-up at a self-selected comfortable speed [20,21]. After familiarization, participants completed 3 sets of 1 min at 5, 10, and 15 km·h^−1^. The last 30 s were used for recording with both systems. Participants were instructed to refrain from strenuous activity for at least 48 h prior to data collection.

### 2.2. Participants

A group of 16 healthy young male adults (age = 22.7 ± 2.6 years; body mass = 69.1 ± 11.7 kg; height = 1.72 ± 0.10 m; weekly training = 6.9 ± 2.4 h/week) participated in the study. All subjects were required to meet the following inclusion criteria: (i) be between 18 and 30 years of age, (ii) not have suffered any injury within six months prior to data collection, and (iii) be physically active according to the guidelines of the American College of Sports Medicine (ACSM) [22]. All the subjects were informed of the purpose and procedures of the study before signing a written consent form. The study protocol adhered to the tenets of the Declaration of Helsinki and was approved by the Institutional Review Board (No. 2546/CEIH/2022).

### 2.3. Procedures

The height (m) and body mass (kg) of the participants were obtained using the stadiometer SECA 222 (SECA, Corp., Hamburg, Germany) and the bioimpedance meter Inbody 230 (Inbody Seúl, Corea), respectively.

**Figure 1:**
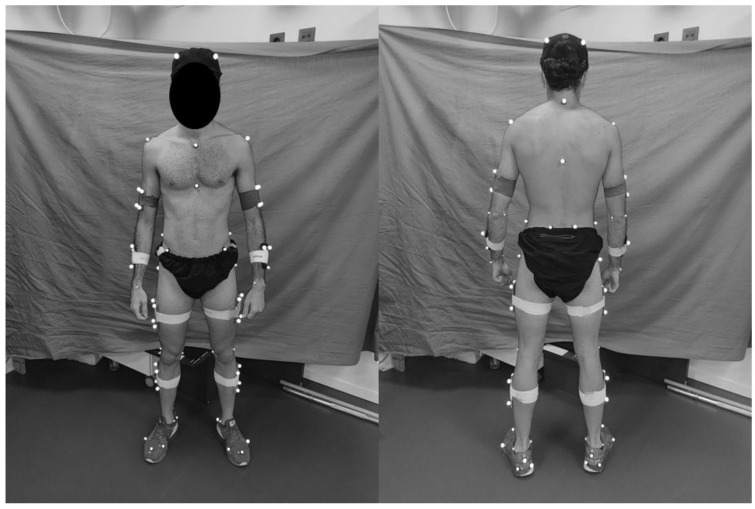
Illustration of the marker set.

Pelvic kinematics were assessed using two different systems (i.e., optoelectronic motion capture system versus IMU). Three-dimensional kinematics were acquired using an eight-camera motion analysis system (Qualisys Medical AB, Sweden) with a sampling rate of 250 Hz. The cameras were positioned to provide a complete view of the treadmill location. Safety bars were removed to avoid any potential masking of the markers by the structures. Prior to the data collection, the volume of the test space was calibrated using a dynamic T-wand, and the origin and axes of the coordinate system were established by placing an L-frame on the treadmill. Subjects were then fitted with a lower body marker model. A total of 40 markers were placed by two experienced researchers based on the palpation of appropriate anatomical landmarks (Figure 1). One of the researchers was responsible for placing the reflective markers and the other researcher was responsible for checking their placement, thus providing a double check. The anatomical locations of the markers were the right and left iliac crest tubercle, the right and left posterior superior iliac spine, the right and left femur greater trochanter, the right and left anterior superior iliac spine, the right and left femur lateral epicondyle, the right and left femur medial epicondyle, the right and left fibula apex of the lateral malleolus, the right and left tibia apex of the medial malleolus, the right and left fifth metatarsal head, the right and left first metatarsal head, and the right and left posterior surface of the calcaneus. In addition, two cluster marker sets (a group of four retro-reflective markers attached to a lightweight rigid plastic shell) were also placed on the thigh and on the shank. Once in position, a static test was performed with the participants positioned in an anatomical position prior to the start of the treadmill running protocol.

All the static and motion tests were exported to Visual 3D (C-Motion Inc, Boyds, ML, USA). The rigid link model created from the static file was then assigned to all the imported motion files. In particular, the pelvic segment was created according to the CODA model [23]. The motion files were filtered with a fourth-order Butterworth low-pass filter with a cut-off frequency of 8 Hz. The x–y–z Cardan sequence was used to calculate joint angles. This sequence corresponds to flexion/extension, abduction/adduction, and axial rotation. Joint angles were not normalized to the static standing test. The laboratory frame followed the right-hand rule and had the positive y-direction oriented in the direction of forward progression, the positive x-direction oriented to the left, and the positive z-direction oriented vertically upward. Vertical oscillation, tilt, obliquity, rotational range of motion, and maximal angular rates were calculated over the entire gait cycle.

**Figure 2:**
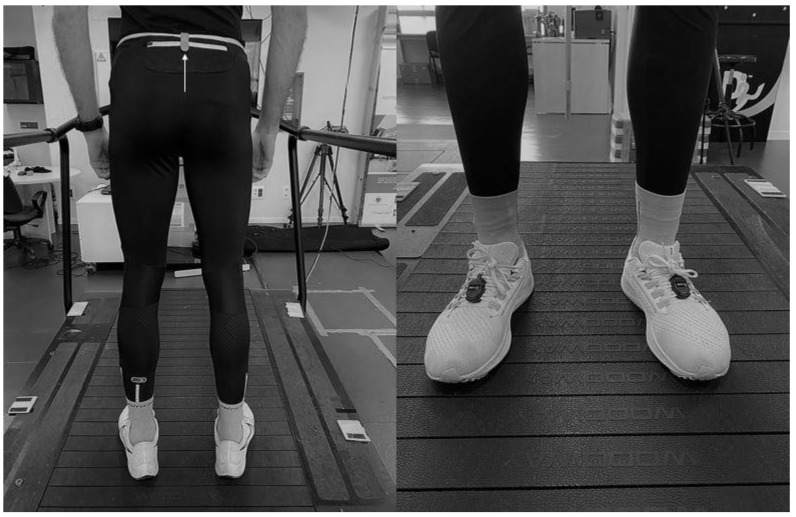
RunScribe sensor placement: **left** panel, sacral sensor and **right** panel, footpods sensors.

The three-sensor Runscribe Sacral Gait LabTM IMU (Scribe Lab. Inc. San Francisco, CA, USA) conducted recording at 500 Hz. This IMU combines an accelerometer, a gyroscope, and a triaxial magnetometer. Following the recommendations of García-Pinillos et al. [18], two RunScribe ™ devices were attached to the laces of the running shoes. A third RunScribe™ device was attached to the waistband of the pants at the height of the sacrum (Figure 2), following the recommendations of the manufacturer. Before data collection, the system was calibrated flat and once mounted according to the manufacturer´s instructions. The pelvic kinematics (i.e., vertical oscillation, tilt, obliquity, rotational range of motion, and maximal angular rates) were collected by the IMU and were then synchronized to the RunScribe platform (https://dashboard.runscribe.com/ accessed on 3 november 2022) where these metrics are reported automatically. From there, they are copied to an Excel sheet for subsequent analysis. 

### 2.4. Statistical Analyses

Descriptive data are presented as mean ± standard deviation (SD) with 95% confidence intervals (CI). The normal distribution of the data and homogeneity of variances were confirmed through the Shapiro–Wilk test and Levene’s tests, respectively. The level of agreement of the RunScribe Sacral Gait Lab™ with the optoelectronic motion capture system was examined through systematic bias, Pearson’s correlation coefficient (r), the standard error of the estimate (SEE) obtained from the linear regression analysis, and the intraclass correlation coefficient (ICC) using a two-way mixed model for absolute agreement [24]. An acceptable level of agreement was considered if the following criteria were met: a low bias and SEE (<0.2 times the between-subjects differences SD) [25], almost perfect (r > 0.90) [26], and good reliability (ICC > 0.81) [24]. Statistical analyses were performed using the software package SPSS (IBM SPSS version 25.0, Chicago, IL, USA). Statistical significance was set at *p* ≤ 0.05.

## 3. Results

### 3.1. Vertical Oscillation

Subjects exhibited a vertical oscillation of 4.7 (1.2), 10.0 (1.6), and 8.2 (1.1) cm at 5, 10, and 15 km/h, respectively (Table 1). The IMU system did not reach the validity criteria established at any of the velocities tested.

### 3.2. Pelvic Tilt

Subjects exhibited a pelvic tilt ROM of 6.8 (2.4), 6.7 (2.0), and 10.2 (3.9)° and a maximal tilt rate of 49.4 (10.4), 126 (53), and 167 (48) °/sec at 5, 10, and 15 km/h, respectively. The IMU system did not reach the validity criteria established at any of the velocities tested. 

### 3.3. Pelvic Obliquity

Subjects exhibited a pelvic obliquity ROM of 9.5 (2.3), 14.5 (4.7), and 22.4 (7.2)° and a maximal obliquity rate of 46.2 (13.5), 141 (31), and 166 (31) °/sec at 5, 10, and 15 km/h, respectively. The IMU system did not reach the validity criteria established at any of the velocities tested. 

### 3.4. Pelvic Rotation

Subjects exhibited a pelvic rotation ROM of 7.9 (2.0), 17.2 (5.2), and 22.9 (7.8)° and a maximal rotation rate of 67.3 (15.2), 128 (29), and 167 (39) °/sec at 5, 10, and 15 km/h, respectively. The IMU did not reach the validity criteria established at any of the velocities tested.

## 4. Discussion

This purpose for this study was to examine the validity of the three-sensor RunScribe Sacral Gait Lab™ to measure pelvic kinematics in terms of vertical oscillation, tilt, obliquity, rotational range of motion, and maximal angular rates during walking and running on a treadmill at 5, 10, and 15 km/h. The results revealed that the IMU did not reach the validity criteria established for any of the variables and velocities tested. 

To the authors’ knowledge, this is the first study to analyze the validity of the RunScribe Sacral Gait Lab™ IMU for measuring pelvic kinematics during human locomotion. As noted above, the potential of using IMU to assess gait analysis without the limitations of laboratory technology is well-known [18]. This is a step forward in terms of trainers and clinicians being able to measure athletes or clients in a natural environment and in a time-efficient manner. However, this advantage would be useless if the data were invalid. The results obtained here indicate that the RunScribe Sacral Gait Lab™ IMU is not valid for measuring pelvic kinematics during walking or running. Similar results have been observed regarding its reliability [27]. However, it is well established that the spatiotemporal parameters reported by this device are valid and reliable [18,19,28]. 

Several earlier studies have analyzed the validity of IMUs in measuring pelvic kinematics during gait and running and concluded that errors greater than 5° could mislead the clinical and performance interpretation [10,28]. Considering other statistical criteria, Bolink et al. [28] found a reasonably satisfactory agreement of the range of movement measurements between both an IMU and an optoelectronic motion capture system, as the deviations of the measured angles were within the limits of agreement of the Bland–Altman plots. Very large correlations and almost perfect ICCs were obtained during gait with respect to pelvic kinematics (i.e., tilt, obliquity, and rotation). As in the present study, Bugané et al. [29] compared pelvic kinematics in three planes during gait using a sacrum mounted IMU. They obtained an error of less than 3° in both the sagittal and frontal planes and nearly perfect correlations. The validity criteria established here were similar to those reported in previous studies, indicating that the IMU may be a valid device for measuring pelvic kinematics, but further improvements to the RunScribe Sacral Gait Lab™ should be considered to achieve the precision required for its use in clinical and performance settings. In addition, the pelvic kinematics were measured in healthy subjects during walking and running at slow and moderate speeds (i.e., 5, 10, and 15 km/h). These were within the normal kinematic ranges as previously reported [2,30]. However, a different level of accuracy may be required in subjects with clinical conditions or athletes tested at higher running speeds.

Regarding the pelvic angular values provided by the RunScribe™ Sacral Gait Lab™, these are based on the peak values. Therefore, the operating range of the accelerometer could be a potential source of error [31]. Another cause that could explain the low validity and reliability [27] of the device could be that it is attached to the waist rather than sports tights, which could potentially introduce more disturbance to the signal.

The main limitation of the study is that the IMU may have been susceptible to motion artifacts due to the mounting method designed by the manufacturer. Another limitation of the work is the software version used as it was recently updated after this study was conducted. In addition, the present study did not analyze the test-retest reliability, which could be considered as a future line of research. Despite these limitations, the current study examines the validity of a commonly used device by trainers and clinicians to analyze lower limb kinematics during human locomotion, which could provide useful information for these professionals.

## 5. Conclusions

The results obtained report significant differences between systems for the pelvic kinematic parameters measured during both walking and running. Therefore, the three-sensor RunScribe Sacral Gait Lab™ has shown a questionable validity according to an optoelectronic motion capture system (250 Hz) for measuring pelvic kinematics in terms of vertical oscillation, tilt, obliquity, rotational range of motion, and maximal angular rates at 5, 10, and 15 km/h.

From a practical point of view, the three-sensor RunScribe Sacral Gait Lab™, as stated above, is a valid device for measuring spatiotemporal parameters [18,27] and foot-strike patterns [15] but shows a low level of agreement with the reference system when measuring pelvic kinematics. Therefore, its use for measuring the aforementioned variables is not recommended. 

## Figures and Tables

**Table 1:** Level of agreement of the pelvic kinematic parameters obtained through the RunScribe Sacral Gait Lab™ and the optoelectronic motion capture system.

	RunScribe	Qualisys	Bias (95% CI)	SEE	SWC	ICC (95% CI)	r
**5 km/h**							
Vertical oscillation (cm)	25.9 (2.0)	4.7 (1.2)	21.1 (20.0 to 22.3)	4.9	0.2	0.01 (−0.01 to 0.07)	0.458
Obliquity ROM (°)	8.4 (1.8)	9.5 (2.3)	−1.1 (−2.9 to 0.6)	3.2	0.5	−0.10 (−1.97 to 0.63)	−0.056
Tilt ROM (°)	7.0 (1.7)	6.8 (2.4)	0.2 (−1.7 to 2.3)	3.3	0.5	−1.62 (−12.5 to 0.25)	−0.429
Rotation ROM (°)	8.6 (2.9)	7.9 (2.0)	0.7 (−0.4 to 1.7)	1.9	0.4	0.83 (0.46 to 0.95)	0.758 *
Obliquity max rate (°/sec)	75.9 (14.6)	46.2 (13.5)	29.7 (18.8 to 40.4)	35	2.7	0.07 (−0.21 to 0.46)	0.121
Tilt max rate (°/sec)	52.1 (16.6)	49.4 (10.4)	2.7 (−8.9 to 14.4)	18	2.1	0.22 (−2.05 to 0.78)	0.164
Rotation max rate (°/sec)	47.1 (8.7)	67.3 (15.2)	−20.2 (−27.9 to −12.5)	25	3.0	0.34 (−0.24 to 0.75)	0.543
**10 km/h**							
Vertical oscillation (cm)	9.6 (2.2)	10.0 (1.6)	−0.4 (−1.3 to 0.3)	1.6	0.3	0.81 (0.47 to 0.94)	0.724 *
Obliquity ROM (°)	9.2 (3.4)	14.5 (4.7)	−5.3 (−7.7 to −2.9)	6.8	0.9	0.44 (−0.28 to 0.80)	0.522
Tilt ROM (°)	11.3 (5.6)	6.7 (2.0)	4.6 (1.8 to 7.2)	7.2	0.4	0.28 (−0.36 to 0.69)	0.389
Rotation ROM (°)	11.1 (3.0)	17.2 (5.2)	−6.1 (−11.8 to −4.9)	11	1.0	−0.32 (−1.12 to 0.40)	−0.312
Obliquity max rate (°/sec)	206 (92)	141 (31)	65 (16 to 114)	105	6.2	0.37 (−0.39 to 0.78)	0.533
Tilt max rate (°/sec)	367 (180)	126 (53)	241 (143 to 339)	320	10.6	0.03 (−0.30 to 0.44)	0.085
Rotation max rate (°/sec)	136 (40)	128 (29)	8.1 (−23 to 39)	57	5.8	−0.50 (−4.66 to 0.54)	−0.198
**15 km/h**							
Vertical oscillation (cm)	5.8 (1.5)	8.2 (1.1)	−2.4 (−3.0 to 1.8)	2.8	9.0	0.37 (−0.18 to 0.77)	0.638 *
Obliquity ROM (°)	16.3 (5.7)	22.4 (7.2)	−6.1 (−9.6 to −2.7)	8.4	1.4	0.577 (−0.22 to 0.86)	0.590 *
Tilt ROM (°)	13.4 (5.3)	10.2 (3.9)	3.2 (−0.2 to 6.6)	7.0	0.8	0.09 (−1.04 to 0.65)	0.056
Rotation ROM (°)	11.7 (3.2)	22.9 (7.8)	−11.2 (−16.6 to −5.8)	15	1.6	−0.30 (−0.79 to 0.39)	−0.481
Obliquity max rate (°/sec)	269 (95)	166 (31)	103 (58 to 148)	138	6.2	0.35 (−0.27 to 0.75)	0.573 *
Tilt max rate (°/sec)	388 (172)	167 (48)	221 (131 to 311)	291	9.6	0.13 (−0.25 to 0.55)	0.333
Rotation max rate (°/sec)	186 (74)	167 (39)	19 (−21.1 to 59.0)	78	7.8	0.39 (−0.77 to 0.79)	0.296

ROM: Range of motion. CI: Confidence interval. SEE: Standard error of the estimate. SWC: Smallest worthwhile change. ICC: Intraclass correlation coefficient. r: Pearson correlation coefficient. *: Correlation is significant at the 0.05 level.

## Data Availability

Data will be available on request.
